# Torsional Deformity Significantly Impacts Lateral Ankle Radiographic Imaging Parameters

**DOI:** 10.7759/cureus.59292

**Published:** 2024-04-29

**Authors:** Matthew J Folkman, Kouami Amakoutou, Asha Ravichandran, Dre’Marcus Ferrell, David M Wang, Bryan O Ren, Alexander Rascoe, Raymond W Liu

**Affiliations:** 1 Pediatric Orthopaedics, The University of Toledo College of Medicine and Life Sciences, Toledo, USA; 2 Pediatric Orthopaedics, Rainbow Babies & Children's Hospital, Cleveland, USA; 3 Pediatric Orthopaedics, Case Western Reserve University School of Medicine, Cleveland, USA; 4 Dermatology, Brigham and Women’s Hospital, Boston, USA; 5 Orthopaedic Surgery, University of Michigan, Ann Arbor, USA; 6 Orthopaedic Surgery, University Hospitals Cleveland Medical Center, Cleveland, USA

**Keywords:** ankle imaging parameters, lateral ankle imaging, tibial torsion, ankle imaging, limb deformity

## Abstract

Background

Optimal lateral ankle imaging is important for the diagnosis and treatment of multiple ankle conditions. The effects of limb deformity on lateral ankle imaging are not well described and are clarified in this osteological study.

Materials and methods

We utilized an osteological collection and imaged all specimens after the first positioning of the talus in the lateral position and positioning the tibia and fibula to match. We then measured the relative positions of the tibia and fibula and their widths to calculate standard ratios. All measurements were evaluated for reliability using intra-class correlation coefficients. Multiple regression analysis determined how patient characteristics, tibial torsion, and medial proximal tibial angle affected various lateral ankle imaging ratios.

Results

The intra-class correlation coefficient was excellent for all measurements. In the multiple regression analysis, all five imaging ratios had at least one statistically significant outcome. The anterior tibiofibular interval (ATFI)-tibial width (TW) ratio (ATFI:TW) had only one association with sex and had the lowest standard deviation. All other parameters had variation with tibial torsion and/or medial proximal tibia angle (MPTA). The mean ATFI was 1.06 ± 0.21 cm and 1.19 ± 0.23 cm for females and males, respectively.

Conclusions

Patient sex and tibial torsion impacted the fidelity of lateral imaging parameters. ATFI:TW may pose the greatest utility given its minimal association with deformity parameters and low standard deviation.

## Introduction

Radiographic parameters of the ankle are of great importance when evaluating ankle injuries and their treatment. Lateral imaging of the ankle has utility in a multitude of clinical presentations. Accurate and optimal lateral imaging of the ankle is critical for the diagnosis, prognosis, and follow-up of treatment in syndesmotic injuries, pilon fractures, fracture-dislocations of the ankle, proper ankle reduction, and ankle osteoarthritis [1-7]. Proper lateral ankle imaging is also critical to determining various angle measurements for optimal surgical planning and correction of lower limb deformities [8,9].

While parameters for ideal anteroposterior (AP) and mortise views are more frequently reported, the parameters defining an ideal lateral ankle radiograph are less clearly defined [2,3,10-14]. The majority of studies that have evaluated the radiographic qualities of the lateral ankle used talar dome overlap to signify a perfect lateral image [2,11,12,15-17]. While this may be an effective method to confirm a quality lateral view when the talar dome is perfectly overlapped, it is hard to quantify how imperfect a view might be. Some radiological studies have evaluated osseus ratios to determine the relative positioning of the tibia and fibula on lateral imaging [2-4], but there remains some variation in measurement methodology and study design [2,4,15,18]. Limitations of previous studies have included homogeneity of patient demographics, lack of interobserver measurement reliability, and limited evaluation of the impact of any associated osseus deformity [18]. In particular, the influence of tibial torsion and coronal plane proximal tibial alignment on all of these parameters is sparsely published.

To clarify these questions, we designed a cadaveric study identifying the position of the fibula versus the tibia on an ideal lateral view. In addition, we investigated if significant variations among patient characteristics, such as demographics and tibial deformity, are associated with changes in lateral ankle imaging measurements.

## Materials and methods

We performed a cadaveric study using specimens from the Hamann-Todd Osteological Collection. The collection contains disarticulated human remains who died in Cleveland, Ohio, primarily between 1910 and 1939. Subject information is organized in a database. We included subjects aged 22-84 years at the time of death. We excluded subjects with incomplete skeletons, fractures, or signs of metabolic disorders, rheumatologic diseases, or infection.

**Figure 1:**
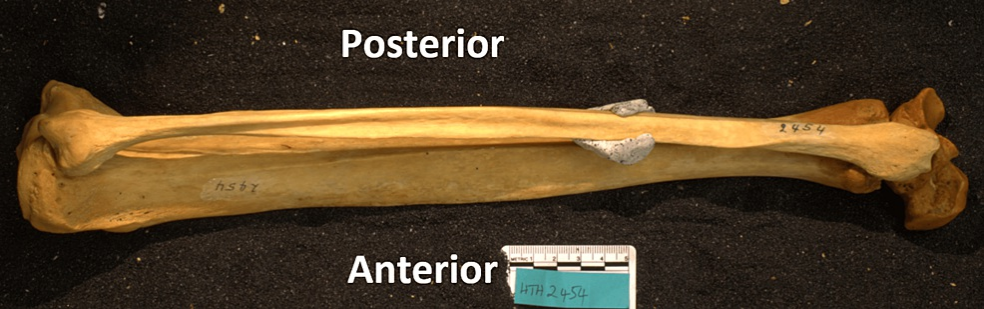
Sample specimen positioning for imaging Sample image positioning of a specimen for image measurement. The use of the sandbox allows for precise and stable positioning of the three bones.

Upon identifying specimens for the study, two authors (blinded for review) positioned the bones of the ankle to replicate the native anatomy and optimal position for imaging. Optimal lateral ankle images were defined as overlapping talar domes with the projection of the fibula on the posterior third of the tibia [4,12,17]. For our experimental setup, the talus was oriented orthogonally to the camera, and then the tibia was positioned to accommodate the shape of the talus. The fibula was then laid on top of the tibia using the proximal and distal tibia-fibula articulations as a guide to complete the joint. All bones of the reconstructed ankle joint were held in place using sand within a transparent display case to allow for proper imaging (Figure 1).

**Figure 2:**
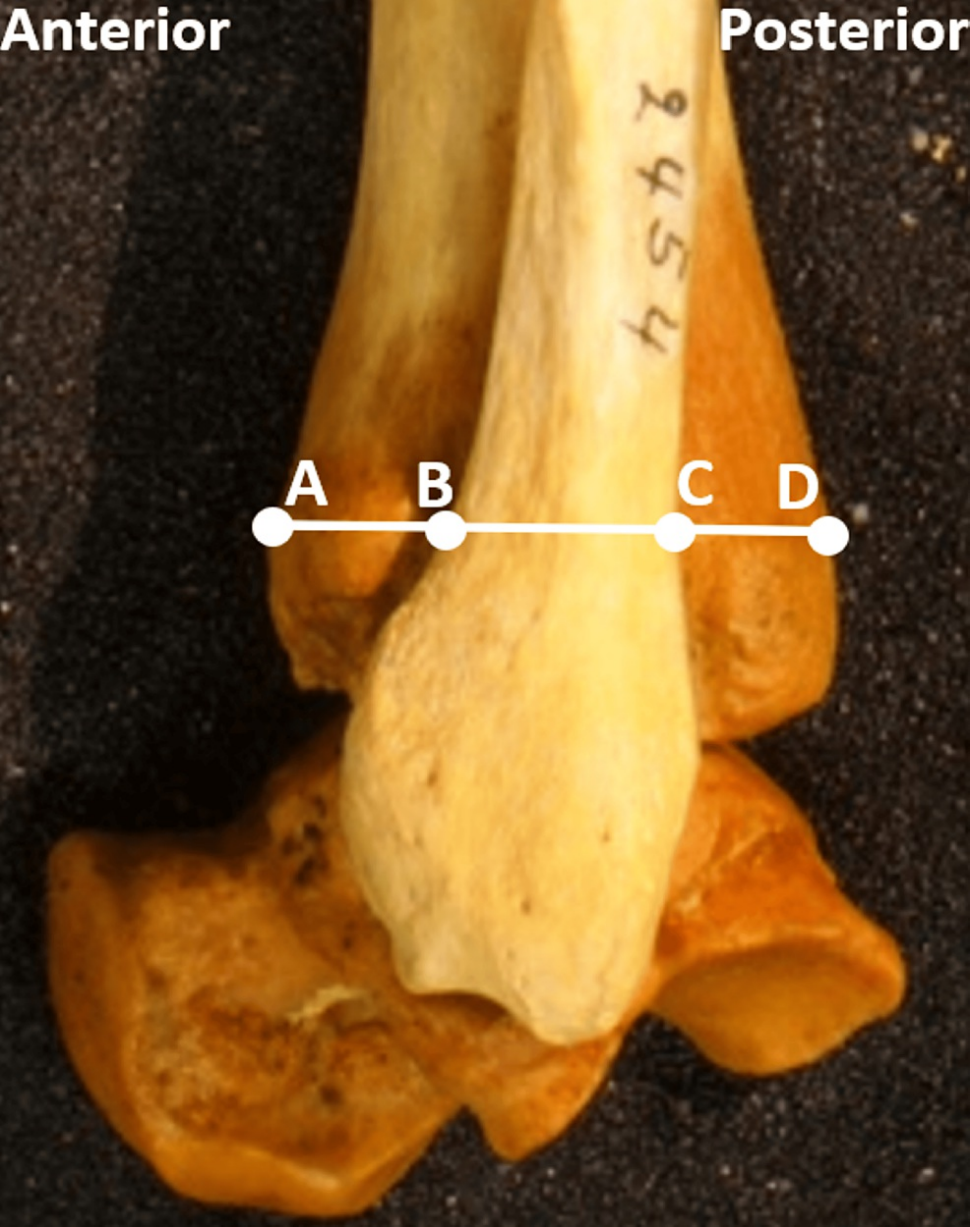
Sample measurement sites of lateral ankle imaging Points A-D represent the tibial width (TW), A-B represent the posterior tibiofibular interval (PTFI), B-C represent the fibular width (FW), and C-D represent the anterior tibiofibular interval (ATFI).

Digital measurements were performed on the captured images. To allow for generalizability among patients and published literature, we used commonly described measurements and ratios [2,4,5,10,19]. Measurements were as follows: tibial width (TW), fibular width (FW), and posterior tibiofibular interval (PTFI) 1 cm above the tibial plafond, as described in similar studies [2]. The PTFI was defined as the distance from the posterior fibular cortex to the posterior tibia. Anterior tibiofibular interval (ATFI) was defined as the distance between the anterior cortex of the tibia to the anterior fibula and calculated using the other measurements (Figure 2) [2]. Two investigators independently positioned, photographed, and measured 20 specimens to test inter-rater reliability. Measurements were evaluated for reliability using an intra-class correlation coefficient (ICC). We used a two-way mixed effects single rater with absolute agreement and two-way mixed effects, absolute agreement, and multiple raters. We performed measurements on two separate occasions repeated three weeks later to test inter and intra-rater reliability. The ICC values were interpreted as follows: a value of <0.5 as poor, 0.5 to 0.75 as moderate, 0.75 to 0.9 as good, and >0.9 as excellent [20].

Our pre-existing database included subject demographics (age, sex, and race) and previously published measurements of tibial torsion and medial proximal tibia angle (MPTA) [21,22]. Patient race was defined based on the original descriptions provided by the anatomic collection. The subject race options at the time of the cadaveric collection were white, black, or other. Tibial torsion was based on the axial plane angle between the anterior edge of the distal tibial plafond and a line that bisected the proximal tibial plateaus [20,21]. The MPTA was defined as the medial angle between mechanical axis of the tibia and the surface of the proximal tibia to determine coronal plane deformity [21,23].

A multiple linear regression analysis was performed for each ratio. Dependent variables were the ratios in lateral imaging, and independent variables were patient age, sex, race (black or white), tibial torsion, and MPTA. Statistics were performed using IBM SPSS Statistics for Windows, version 27.0 (released 2020, IBM Corp., Armonk, NY). We considered p < 0.05 to be significant.

## Results

There were a total of 131 subjects, and the demographics are summarized in Table 1. The mean tibial torsion was 4.5 ± 7.9 degrees, and the mean MPTA was 87.3 ± 2.9 degrees. The inter-rater ICC values for the tibial width, fibular width, and posterior tibial fibular distance overlap were 0.99, 0.98, and 0.99, respectively. The intra-rater ICC values for the same parameters were 0.99, 0.96, and 0.99, respectively, indicating excellent agreement. Tibia and fibula measurement and ratio means are summarized in Table 4.

**Table 1:** Summary of patient demographics (sex and race)

	n = 131	%
Male	116	89%
Female	15	11%
White	78	60%
Black	53	40%

All five regression models produced at least one significant association for patient demographics and deformity parameters with lateral radiographic ratios (Table 2 and Table 3). The ATFI:TW ratio had only one association with sex and had the lowest standard deviation. Conversely, the other models had multiple statistically significant associations and/or were significantly associated with tibial torsion or the MPTA. Among patient sex, the mean ATFI was 1.06 ± 0.21 cm and 1.19 ± 0.23 cm for females and males, respectively. The FW:TW ratio had four significant associations, the most in this study.

**Table 2:** Multiple regression analysis for measurement ratios and characteristics Multiple regression results listing standardized β values organized by interval ratio and demographics. * indicates significance of p < 0.05. ATFI: anterior tibiofibular interval, TW: tibial width, PTFI: posterior tibiofibular interval, FW: fibular width, MPTA: medial proximal tibia angle

	ATFI:TW		PTFI:TW		PTFI:(PTFI+FW)		ATFI:(ATFI+FW)		FW:TW	
Characteristic	β	p	β	p	β	p	β	p	β	p
Age	0.03	0.87	-0.15	0.36	-0.16	0.29	-0.05	0.76	0.12	0.36
Sex	-0.44	0.03*	-0.05	0.79	-0.16	0.33	-0.48	0.01*	0.40	0.01*
Race	0.26	0.20	0.09	0.63	0.14	0.40	0.30	0.08	-0.29	0.05*
Tibial torsion	-0.03	0.87	-0.42	0.02*	-0.48	0.01*	-0.24	0.14	0.41	0.01*
MPTA	-0.26	0.15	-0.14	0.41	-0.20	0.19	-0.32	0.04*	0.33	0.01*

**Table 3:** Measurement ratios with 95% confidence intervals and correlation coefficients with unstandardized beta values The upper and lower bounds of the 95% confidence intervals and the R^2^ values for multiple linear regression analysis. ATFI: anterior tibiofibular interval, TW: tibial width, PTFI: posterior tibiofibular interval, FW: fibular width, MPTA: medial proximal tibia angle

	ATFI:TW			PTFI:TW			PTFI:(PTFI+FW)			ATFI:(ATFI+FW)			FW:TW		
Characteristic	Lower	Upper	R^2^	Lower	Upper	R^2^	Lower	Upper	R^2^	Lower	Upper	R^2^	Lower	Upper	R^2^
Age	-0.01	0.00	0.03	-0.00	0.00	-0.17	-0.00	0.00	-0.19	-0.00	0.00	-0.06	-0.00	0.00	0.17
Sex	-0.18	-0.01	-0.39	-0.01	0.08	-0.05	-0.15	0.05	-0.17	-0.21	-0.04	-0.48	0.03	0.18	0.47
Race	-0.01	0.07	0.23	-0.03	0.05	0.01	-0.03	0.07	0.15	-0.00	0.01	0.31	-0.07	-0.00	-0.35
Tibial torsion	-0.00	0.00	-0.29	-0.01	0.00	-0.40	-0.01	-0.00	-0.48	-0.00	0.00	-0.27	0.00	0.01	0.48
MPTA	-0.01	0.00	-0.25	-0.01	0.00	-0.15	-0.01	0.00	-0.23	-0.01	0.00	-0.36	0.00	0.01	0.43

## Discussion

We performed a cadaveric study to assess the optimal positioning of the fibula relative to the tibial on lateral ankle imaging and its association with deformity parameters. The ATFI:TW ratio demonstrated only one association with sex, with the lowest standard deviation among measured parameters.

Three of our lateral imaging regression models, FW:TW, ATFI:TW, and ATFI:(ATFI+FW), were significantly associated with patient sex. Differences in lateral ankle radiographic imaging results among patient sex are not uncommon, and similar results have been reported in the literature [3,10,24], although a lack of association has also been reported [4]. Despite reported measurement variations with sex, some studies still concluded these were the most effective measurements for assessing the lateral ankle [2,3]. In contrast to our results, a study by Croft et al. reported that the use of the ATFI:TW ratio produced outcomes that best controlled for patient sex and was their recommended lateral imaging parameter [2].

While our study did not find any association with age, similar studies found patient age significantly impacted lateral ankle imaging parameters [10,24]. This may be due to ligamentous contributions that potentially could change with age. Since our study used disarticulated skeletons, soft tissue assessment was not possible.

Other studies have also explored measurement fidelity between left and right ankles [12,24]. Researchers in these studies concluded there was no significant difference in the measurements of lateral radiograph parameters between the laterality of ankles [12,24].

Several studies have evaluated the ankle joint for optimal imaging, but variability exists in reported measurements. We compared our measurement results to similarly designed studies in Table 4. We found that our interval measurements were relatively consistent with other studies from the published literature, although some variation existed among all studies [2,5,12].

**Table 4:** Lateral ankle radiographic parameters in published studies Mean lateral radiographic measurements and ratios compared to other reported values in similar published studies. ATFI: anterior tibiofibular interval, TW: tibial width, PTFI: posterior tibiofibular interval, FW: fibular width

	Present study		Croft et al. [2]		Yaradılmış et al. [5]		Kellam et al. [12]	
Measurements	Mean	SD	Mean	SD	Mean	SD	Mean	SD
Tibia width (cm)	4.26	0.24	-	-	-	-	-	-
Posterior T-F (cm)	1.03	0.28	-	-	0.61	0.29	-	-
Fibula width (cm)	2.06	0.31	-	-	-	-	-	-
Anterior T-F (cm)	1.17	0.23	-	-	1.3	0.24	-	-
Ratios								
ATFI:TW	0.28	0.05	0.39	0.09	0.4	0.1	0.29	0.06
PTFI:TW	0.24	0.06	0.17	0.06	-	-	0.15	0.05
PTFI:(PTFI + FW)	0.33	0.08	0.27	0.06	-	-	-	-
ATFI:(ATFI + FW)	0.36	0.06	0.46	0.07	-	-	-	-
FW:TW	0.48	0.07	-	-	-	-	0.52	0.08

While researchers have explored the impact of rotational patient positioning on lateral and AP imaging studies, few have explored the effects of tibial torsion on imaging [3,25,26]. This is likely due to the need for full-length tibial computed tomography (CT), magnetic resonance imaging (MRI), or low-dose radiographs (e.g., EOS images) to quantify tibial torsion. We found that tibial torsion was significantly associated with ratio measurements from two regression models, PTFI:TW and PFTI:(PTFI+FW), suggesting that PTFI may not be the best parameter to quantify the quality of a lateral ankle radiograph. Although three-dimensional CT or MRI may help minimize measurement errors found with lateral ankle radiographs, their use is not practicable or affordable in many practices and is generally not easily available intraoperatively.

While this study provides new insight into optimal lateral ankle imaging characteristics, it is not without its limitations. There was a limited female sex population in this study, which may limit its generalizability to other populations. The ankles in this study were disarticulated and required positioning by sight, which had potential inaccuracy. However, the excellent ICC values between the raters each separately positioning the specimens support the reproducibility of our methodology. In addition, our measurements fit well when compared to the literature. Nevertheless, the lack of ligamentous structures may affect the validity of the measurements. Given this, there is likely more value in the associations between age and deformity found in this study, rather than the raw values.

## Conclusions

Our data suggest that the ATFI:TW ratio is best for the evaluation of lateral ankle imaging. The ratio has resilience to variations in tibial torsion and the MPTA, and its low standard deviation suggests that it is the most consistent option among standard ratios. Future clinical studies are necessary to determine its full utility.
